# Determination of the ability of matrix-assisted laser desorption ionization time-of-flight mass spectrometry to identify high-biofilm-producing strains

**DOI:** 10.3389/fmicb.2022.1104405

**Published:** 2023-01-10

**Authors:** David Rodríguez-Temporal, Rafael Díez, Marta Díaz-Navarro, Pilar Escribano, Jesús Guinea, Patricia Muñoz, Belén Rodríguez-Sánchez, María Guembe

**Affiliations:** ^1^Department of Clinical Microbiology and Infectious Diseases, Hospital General Universitario Gregorio Marañón, Madrid, Spain; ^2^Instituto de Investigación Sanitaria Gregorio Marañón, Madrid, Spain; ^3^School of Biology, Universidad Complutense de Madrid, Madrid, Spain; ^4^School of Medicine, Universidad Complutense de Madrid, Madrid, Spain; ^5^CIBER Enfermedades Respiratorias-CIBERES (CB06/06/0058), Madrid, Spain

**Keywords:** biofilm, MALDI-TOF MS, mass spectrometry, classification, crystal violet, *Staphylococcus aureus*, *Candida albicans*

## Abstract

**Background:**

The traditional method for assessing the capacity of a microorganism to produce biofilm is generally a static *in vitro* model in a multi-well plate using the crystal violet (CV) binding assay, which takes 96 h. Furthermore, while the method is simple to perform, its reproducibility is poor.

**Objective:**

We evaluated whether matrix-assisted laser desorption ionization time-of-flight mass spectrometry (MALDI-TOF MS) could make it possible to differentiate between high-and low-biofilm-producing microorganisms on 24-h cultures of *Staphylococcus aureus* and *Candida albicans*.

**Methods:**

We included 157 strains of *S*. *aureus* and 91 strains of *C*. *albicans* obtained from the blood cultures of patients with bacteremia/candidemia. We tested biofilm production using the CV binding assay as the gold standard to classify strains as low or high biofilm producers. We then applied MALDI-TOF MS to create a machine learning–based predictive model using 40 strains of *S*. *aureus* and *C*. *albicans*, each with extreme absorbance values, and validated this approach with the remaining 117 and 51 strains using the random forest algorithm and the support vector machine algorithm, respectively.

**Results:**

Overall, 81.2% of the *S*. *aureus* strains (95/117) and 74.5% of the *C*. *albicans* strains (38/51) used for validation were correctly categorized, respectively, as low and high-biofilm-producing.

**Conclusion:**

Classification based on MALDI-TOF MS protein spectra enables us to predict acceptable information about the capacity of 24-h cultures of *S*. *aureus* and *C*. *albicans* to form biofilm.

## Importance

We provide a new application of MALDI-TOF MS protein spectra that enables us to predict acceptable information about the capacity of 24-h cultures of *S*. *aureus* and *C*. *albicans* to form biofilm.

## Introduction

Biofilm production is a pathogenic characteristic of all microorganisms. It is considered a virulence risk factor and attempts have been made to correlate it with patient outcome but, unfortunately, the clinical significance of this characteristic remains open to debate, as controversial findings have been reported (Høiby et al., 2011; Kwiecinski et al., 2019). Some studies show an association between the presence of high-biofilm-producing strains and worse prognosis, greater severity, and the possibility that the central venous catheter is the focus of the infection, both in bacteremia/candidemia and in other local infections (Vogel et al., 2000; Jeong et al., 2014; Oufrid et al., 2015; Brunetti et al., 2017; Di Domenico et al., 2017; Monfredini et al., 2018). However, other studies, mainly in bacteremia/candidemia, have not been able to corroborate these findings, attributing symptoms to inadequacy of the *in vitro* model used (Martínez et al., 2006; Guembe et al., 2014; Muñoz-Gallego et al., 2017; Guembe et al., 2018; Muñoz et al., 2018).

Biofilm-producing strains can be identified using various models, both *in vitro* and *in vivo*. The *in vitro* model is the most common, with two subtypes, namely, static and dynamic (Lebeaux et al., 2013; Al Kassaa et al., 2019; Guzmán-Soto et al., 2021). Consequently, there is no standard technique or model capable of reproducibly identifying and differentiating between strains according to their biofilm-forming capacity, and the disparate nature of reported results may depend on the material of the multi-well plate used (Díaz-García et al., 2019). Likewise, within the static model on a multi-well plate, which is the most common method, biomass production can be determined using crystal violet staining (CV) and metabolic activity production by tetrazolium salt staining (XTT), which are complementary and not concordant (Marcos-Zambrano et al., 2014; Alonso et al., 2016; Latorre et al., 2020). Finally, the limitation of this technique is that it takes 96 h to perform. On this basis, it is necessary to look for alternative, objective, reproducible, and rapid tools that can be universally applied to identify biofilm-producing strains to optimize patient outcome and thus initiate early treatment.

Matrix-assisted laser desorption ionization time-of-flight mass spectrometry (MALDI-TOF MS) is based on the detection of the protein profile of the microorganism through its species-specific spectra (Croxatto et al., 2012; Patel, 2015). The incorporation of MALDI-TOF MS into the diagnostic routine of microbiology laboratories constitutes a revolutionary advance owing to its ability to identify bacterial and fungal species in minutes, both from pure culture and from clinical samples, significantly speeding up diagnosis and thus improving clinical management of patients (Tsuchida et al., 2020; García-Riestra et al., 2021; Oviaño and Rodríguez-Sánchez, 2021; Torres-Sangiao et al., 2021). Moreover, MALDI-TOF MS is already being used to identify and differentiate between other intra-species characteristics, such as antibiotic resistance profile, association with virulence genes, or even subspecies (Weis et al., 2020, 2022). To date, few studies have examined the ability of MALDI-TOF MS to differentiate between strains according to whether they have high or low biofilm-forming capacity, and available data are limited in terms of the species tested (Mlynáriková et al., 2016; Caputo et al., 2018; De Carolis et al., 2019; Aguiar, 2021). Moreover, no studies have assessed the use of MALDI-TOF MS for determination of biofilm production in bacteria and fungi from patients with bloodstream infections.

The present study aims to determine the ability of MALDI-TOF MS to classify strains according to their capacity for biofilm production (high or low) compared with traditional biofilm determination based on CV staining.

## Materials and methods

We retrospectively selected a total of 248 strains, 157 of which were *Staphylococcus aureus* (Supplementary Table S1) and 91 *Candida albicans* (Supplementary Table S2). Strains were isolated from blood cultures of patients with bloodstream infections between 2012 and 2015. All isolates were previously identified by MALDI-TOF MS and their antibiotic susceptibility profile was determined using the broth microdilution method Microscan® System (Beckman-Coulter, CA, United States) applying EUCAST (2021) criteria. Moreover, the isolates were also tested for biofilm production. Then, they were kept at −80°C in skimmed milk for further analysis.

We selected *S*. *aureus* and *C*. *albicans* strains isolated from patients with bacteremia, as they are the most virulent and difficult-to-treat microorganisms in which biofilm production in catheter surface is almost the cause of the bacteremia.

### 96-well plate biofilm method

We tested biofilm production using the static 96-well plate method (gold-standard). An inoculum was prepared from 24-h cultures by inoculating several colonies in 50 mL of enrichment medium in Falcon tubes (tryptic soy broth for *S*. *aureus* and yeast peptone dextrose for *C*. *albicans*). The inoculum was then incubated with orbital shaking (165 rpm) at 37°C for 24 h. The tubes were then centrifuged at 1,500 rpm for 5 min and washed three times with PBS (pH = 7.4). We used the resulting suspension to prepare a suspension containing 10^8^ cfu/mL (0.5 McFarland) for *S*. *aureus* and 10^6^ cfu/mL (0.35 McFarland) for *C*. *albicans*. The polystyrene 96-well plate (Greiner Bio-One España S.A.U., Madrid, Spain) was inoculated with 100 μL of each suspension and incubated at 37°C for 24 h. The medium was then removed from the wells, and the plate was washed 3 times with PBS (pH = 7.4) and left to dry. Finally, CV staining was performed to measure biomass in a spectrophotometer (Biochrom EZ Read 400, Biochrom Ltd., Cambridge, United Kingdom), with absorbance read at 550 nm (Vandecandelaere et al., 2016).

We classified strains into tertiles in ascending order according to their absorbance values obtained by the CV binding assay (Guembe et al., 2018; Alonso et al., 2022). Thus, absorbance values for *S*. *aureus* were classified as high (≥1.292), moderate (0.530–1.291), and low (<0.530); those for *C*. *albicans* were also classified as high (≥1.428), moderate (0.922–1.427), and low (<0.922).

### Matrix-assisted laser desorption ionization time-of-flight mass spectrometry

MALDI-TOF MS was performed on 24-h-cultured isolates (Columbia blood agar [bioMérieux, France] for *S*. *aureus* and Candida Chrom-agar [Chrom-agar, United States] for *C*. *albicans*) based on a previous pilot study using 40 strains of *S*. *aureus* in which strains were equally classified using either 24-h-cultured or resuspended sessile cells grown in wells once biofilm had formed (data not shown). A colony of each strain was spotted on two positions of the MALDI-TOF MS plate and covered with 1 μL of 100% formic acid in order to lyse the cells and allow them to dry at room temperature. Then, 1 μL of organic matrix (α-cyano-hydroxycinnamic acid [HCCA, Bruker Daltonics, Bremen, Germany]) was added and allowed to dry. The plate was analyzed using an MBT Smart MALDI Biotyper (Bruker Daltonics) in the range between 2,000 and 20,000 Daltons.

### Spectra analysis

The MALDI-TOF MS plate was read twice, and spectra were acquired using default settings. They were then processed and further analyzed using Clover MS Data Analysis software (Clover Biosoft, Granada, Spain) in the following steps: (1) Variance stabilization; (2) Smoothing by Savitzky–Golay filter with window length = 11 and polynomial order = 3; (3) Baseline subtraction (top-hat filter = 0.02); and (4) Normalization by total ion current. To determine agreement between the gold standard and MALDI-TOF MS, we selected 40 strains of each species classified according to the above tertiles as high-biofilm-producing (*n* = 20) or low-biofilm-producing (*n* = 20) to generate predictive models. We applied the machine learning supervised algorithms partial least squares-discriminant analysis (PLS-DA) and random forest plot (RF). In order to validate the model, we subsequently included the remaining 117 *S*. *aureus* strains (58 high-and 59 low-biofilm-producing) and 51 *C*. *albicans* strains (25 high-and 26 low-biofilm-producing). ROC curves representing the probability of identification obtained for each algorithm were created.

### Ethical statement

Our local ethics committee exempted us from obtaining an inform consent, as it was a retrospective study in which no clinical data were recorded.

## Results

### Staphylococcus aureus

**Figure 1:**
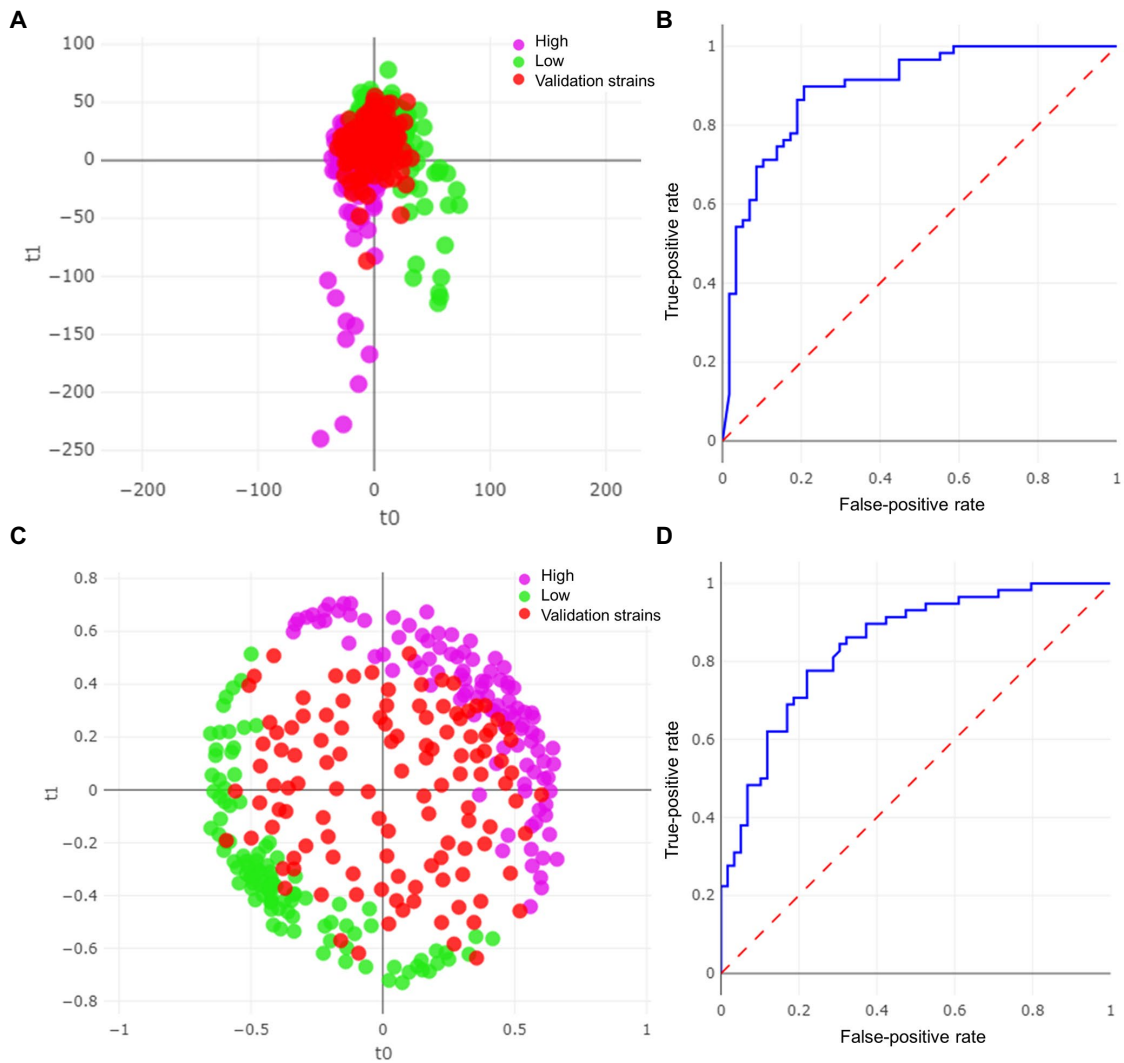
Results of the models for *Staphylococcus aureus* strains. **(A)** Partial least squares-discriminant analysis (PLS-DA) plot with validation strains. **(B)** ROC curve for PLS-DA model. **(C)** Random forest (RF) model with validation strains. **(D)** ROC curve for RF model.

**Figure 2:**
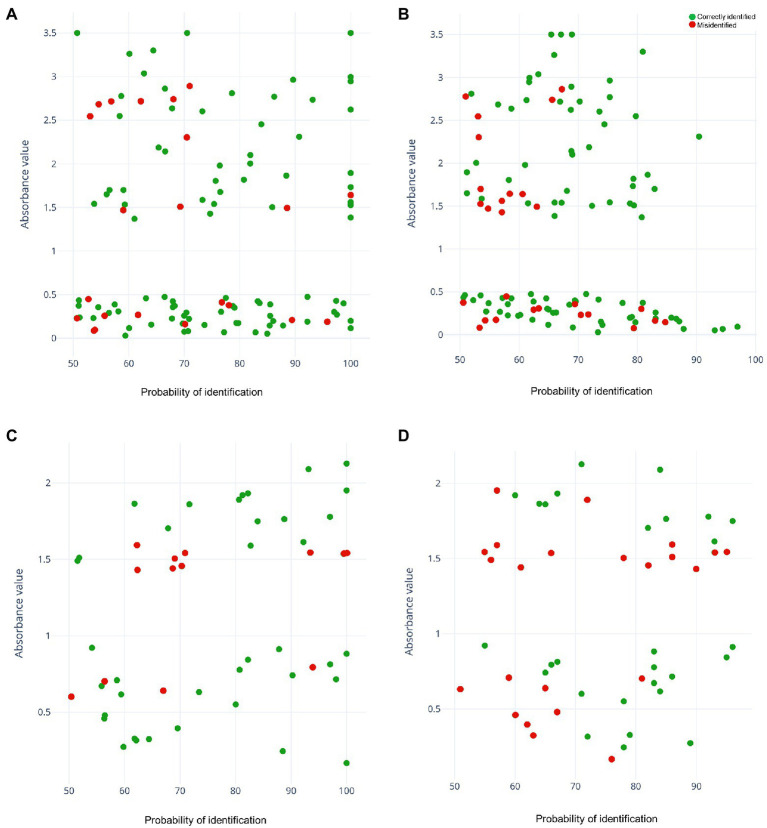
Representation of all strains analyzed according to their absorbance values and the probability of identification obtained using the predictive models. Green dots represent the correctly identified strains and red dots represent misidentified strains. **(A)** Partial least squares-discriminant analysis (PLS-DA) model for *Staphylococcus aureus*. **(B)** Random forest (RF) model for *S*. *aureus*. **(C)** PLS-DA model for *Candida albicans*. **(D)** RF model for *C*. *albicans*.

From the 40 selected strains, we obtained a total of 208 spectra. As this was the first approach, we tested 3 samples/strain and took 2 readings, with 6 experiments/strain (32 had no spectra and were eliminated). k-fold cross-validation yielded higher accuracy with the RF algorithm (97.1%, Table 1). However, the final correlation of the remaining 117 strains used for external validation yielded a higher result with the PLS-DA algorithm (81.2%; Figure 1A) than with the RF algorithm (Figure 1C). Moreover, this difference was observed in the area under the curve (AUC) for the PLS-DA ROC curve (0.895; Figure 1B) compared with an AUC of 0.841 obtained with the RF algorithm (Figure 1D). The green dots represent the low-biofilm-producing strains used for model training (*n* = 20), violet dots represent the high-biofilm-producing strains (*n* = 20), and red dots represent those used to validate the model (117 high-and low-biofilm-producing strains). The same number of strains in each category (low-or high-producing) was misidentified by PLS-DA, and similar numbers were observed for RF (Table 1). Moreover, misidentifications in the PLS-DA algorithm yielded probability values in the range of 50–100% for probability of identification (Figure 2A). Misidentifications in RF were in the range of 50–70% for probability of identification (Figure 2B).

**Table 1:** Identification of *Staphylococcus* *aureus* strains: k-fold cross-validation and external validation.

Actual/predicted	k-fold cross-validation			External validation		
	Low	High	% correct	Low	High	% correct
PLS-DA						
Low	100	5	95.2%	48	11	81.4%
High	7	96	93.2%	11	47	81.0%
**Total PLS-DA**			**94.2%**			**81.2%**
RF						
Low	100	5	95.2%	45	14	76.3%
High	1	102	99.0%	13	45	77.6%
**Total RF**			**97.1%**			**76.9%**

### Candida albicans

**Figure 3:**
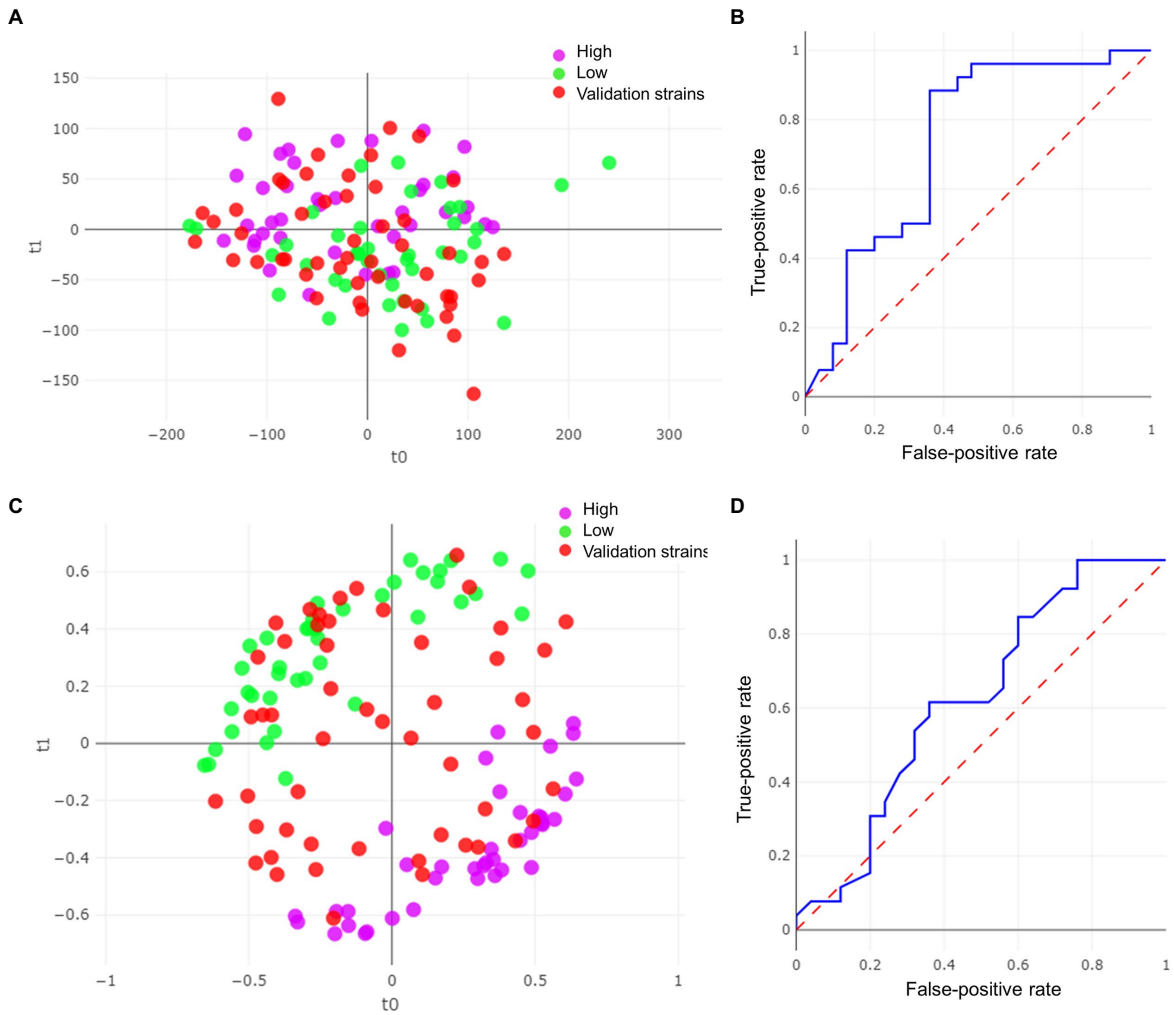
Results of the model for *Candida albicans* strains. **(A)** Partial least squares-discriminant analysis (PLS-DA) plot with validation strains. **(B)** ROC curve for PLS-DA model. **(C)** Random forest (RF) model with validation strains. **(D)** ROC curve for RF model.

We obtained a total of 80 spectra from the 40 strains selected. The RF algorithm yielded a k-fold cross validation accuracy of 92.5% (Table 2). For the validation set, the remaining 51 strains out of the 91 were included and, as observed above, the final correlation was higher with the PLS-DA algorithm (74.5%, Figure 3A) than with the RF algorithm (Figure 3C). In this case, a considerable difference in the area under the ROC curve between the two models was obtained (0.734 for PLS-DA and 0.622 for RF; Figures 3B, D). In both algorithms, the number of misidentifications was greater among high-biofilm-producing strains (Table 2; Figures 2C, D), which were classified as low-biofilm-producing by the models. However, as can be observed in Figure 2C, the misidentified strains were located on the borderline for high-biofilm-producing strains according to their absorbance).

**Table 2:** Identification of *Candida albicans* strains: k-fold cross-validation and external validation.

Actual/predicted	k-fold cross-validation			External validation		
	Low	High	% correct	Low	High	% correct
PLS-DA						
Low	35	5	87.5%	22	4	84.6%
High	12	28	70.0%	9	16	64.0%
**Total PLS-DA**			**78.7%**			**74.5%**
RF						
Low	38	2	95.0%	17	9	65.4%
High	4	36	90.0%	14	11	44.0%
**Total RF**			**92.5%**			**54.9%**

## Discussion

We observed a good correlation between MALDI-TOF MS and the multi-well plate gold standard for classifying high-and low-biofilm-producing strains of *S*. *aureus*. In contrast, the correlation for *C*. *albicans* was moderate.

Since the ability to form biofilm can be an indicator of pathogenicity and is widely used in clinical studies, it would be interesting for laboratory results to become available quickly (Vogel et al., 2000; Klingenberg et al., 2005; Tumbarello et al., 2012; Jeong et al., 2014; Oufrid et al., 2015; Rajendran et al., 2016; Brunetti et al., 2017; Di Domenico et al., 2017, 2019; Monfredini et al., 2018; Tascini et al., 2018). However, traditional methods take at least 96 h and their clinical impact is controversial, mainly owing to the lack of interlaboratory reproducibility of the methods (Guzmán-Soto et al., 2021).

Therefore, alternatives are needed to determine whether a strain is high-or low-biofilm-producing in a more rapid and reproducible manner. In our study, we assessed whether MALDI-TOF MS, which has been shown to be highly capable of identifying for intra-and interspecies differences associated with various factors, could be useful for differentiating between strains according to their ability to produce biofilm. Therefore, we chose the two most virulent species with the worst prognosis for patients, as it is important to clarify the association between biofilm production and patient prognosis (Vogel et al., 2000; Martínez et al., 2006; Guembe et al., 2014; Oufrid et al., 2015; Brunetti et al., 2017; Muñoz-Gallego et al., 2017; Di Domenico et al., 2018; Guembe et al., 2018; Monfredini et al., 2018; Muñoz et al., 2018). Specifically, after analysis of the correlations between MALDI-TOF MS and the gold standard of biofilm staining by CV, both *S*. *aureus* and *C*. *albicans* yielded a high correlation index with the 40 strains used to create the model. However, when the models were validated with the remaining strains, the correlation weakened for the RF algorithm. By contrast, PLS-DA, which was the only linear algorithm applied, yielded the best classification results for both species tested, correctly identifying more than 80% of *S*. *aureus* strains. Likewise, comparison of the results obtained for *S*. *aureus* with those obtained for *C*. *albicans* revealed that the former were even better, as the strains were better categorized, probably because the selected *C*. *albicans* strains classified as high biofilm producers did not show high CV absorbance values (unlike *S*. *aureus*) and may be closer to the “moderate” than to the “high” category. Moreover, since the misidentified strains of *C*. *albicans* had absorbance values on the borderline of high-biofilm-producing strains, they were probably considered low producers by the model. The addition of a higher number of strains with absorbance values covering the complete range could improve the model in the future.

Only four previous studies have compared MALDI-TOF MS with CV staining in *Staphylococcus* strains or yeasts. In the first, MALDI-TOF MS was used to predict biofilm-forming capacity in only 18 strains of *S*. *epidermidis*, revealing a good correlation with respect to CV staining (Caputo et al., 2018). This finding is corroborated in our study in other species such as *S*. *aureus* and *C*. *albicans* and in a significant number of strains. The second study was performed on 113 different *Candida* spp., 107 (95%) of which were high-biofilm-producing (CV absorbance >1.17) and in which the authors demonstrated that MALDI-TOF MS was able to differentiate between specific biofilm proteins, subsequently validating the correlation between 4 high-producing strains and 4 low-producing strains (Aguiar, 2021). We also validated this result using a larger number of strains (51), revealing a correlation of 67.7%. The third study was performed with *Candida parapsilosis*; only 12 high-biofilm-producing and 9 and low-biofilm-producing strains were included. The authors were unable to differentiate between them using MALDI-TOF MS owing to the large variation in the resulting mass spectra (Mlynáriková et al., 2016). In the fourth study, De Carolis et al. (2019) compared mass spectra obtained from attached and suspended isolate cells of 50 *C*. *parapsilosis* clinical strains during the early adhesion phase of *in vitro* biofilm formation using BIOF-HILO, a MALDI-TOF MS assay based on the composite correlation index, to identify differences in mass spectra between the two cell types, classifying strains as high-or low-biofilm-producing. Another study demonstrated the effectiveness of MALDI-TOF MS in differentiating between stages of biofilm formation in *Pseudomonas aeruginosa*, even the point at which cells were released during the dispersion stage (Pereira et al., 2015).

Therefore, we consider that our results are sufficiently robust to propose MALDI-TOF MS as a useful technique for classifying *Staphylococcus* and *Candida* strains according to biofilm production. The procedure is approximately 72 h quicker than the traditional method, thus speeding up and simplifying determination of biofilm production in the microbiology laboratory. The rapid information provided to clinicians by MALDI-TOF MS could improve treatment outcomes in patients with bacteremia or candidemia. The fact that most peaks found in the spectral region of 2,000–20,000 Da are associated with ribosomal proteins, suggests that high-and low-biofilm-producing strains may differ slightly in terms of their proteins, although further studies are needed to characterize them.

Regarding the methodology for spectra classification, different algorithms are applied to a group of data (training set) in order to develop a classification model that will be later tested with a different group of data (validation set). Non-supervised algorithms (Principal Component Analysis or Hierarchical Clustering) might be preferable to supervised algorithms (PLS-DA, SVM, RF, KNN, etc) because they are available to a high number of researchers. In this case, the compared algorithms are both non-supervised. PLS-DA seems to be the fittest algorithm for the classification of the analyzed data. It can be applied by using the software described in the article (Clover MS Data Analysis) or by programming with python or R.

One of the limitations of the study is that this procedure cannot be applied to polimicrobial biofilms, as two different protein spectra would be obtained.

## Conclusion

Our study supports the potential use of MALDI-TOF MS for classification of clinical strains of both *S*. *aureus* and *C*. *albicans* according to their biofilm production. Future studies should expand the number of strains, including new species and additionally using whole genome sequencing. Moreover, reproducibility testing should be assessed to confirm our results.

## Data availability statement

The original contributions presented in the study are included in the article/Supplementary material, further inquiries can be directed to the corresponding author.

## Author contributions

MG and BR-S were responsible for the organization and coordination of the trial. DR-T, MD-N, BR-S, PM, and MG were the chief investigator and responsible for the data analysis. DR-T, MD-N, RD, PE, and JG developed the trial design and data collection. All authors contributed to the writing of the final manuscript.

## Funding

MG was supported by the Miguel Servet Program (ISCIII-MICINN, MSII18/00008) from the Health Research Fund (FIS) of the Carlos III Health Institute (ISCIII), Madrid, Spain. MD-N was supported by the Consejería de Educación, Juventud y Deporte de la Comunidad de Madrid and Fondo Social Europeo (PEJD-2020-AI_BMD-17971). DR-T was supported by the Instituto de Investigación Sanitaria Gregorio Marañón through its intramural program. BR-S was supported by the Miguel Servet Program (ISCIII-MICINN, CPIII19/00002). PE (CPII20/00015) is the recipient of a Miguel Servet contract supported by the FIS. JG is a full-time researcher contracted by Fundación para Investigación Sanitaria del Hospital Gregorio Marañón. The study was partially funded by grants from the Fundación Mutua Madrileña (FMM21/01), ISCIII (PI21/00344), and European Regional Development Fund (FEDER) “A way of making Europe.”

## Conflict of interest

JG has received funds for participating in educational activities organized on behalf of Gilead, MSD, and Pfizer, and also received research funds from FIS, Gilead, Scynexis, F2G, and Cidara, outside the submitted work.

The remaining authors declare that the research was conducted in the absence of any commercial or financial relationships that could be construed as a potential conflict of interest.

## Publisher’s note

All claims expressed in this article are solely those of the authors and do not necessarily represent those of their affiliated organizations, or those of the publisher, the editors and the reviewers. Any product that may be evaluated in this article, or claim that may be made by its manufacturer, is not guaranteed or endorsed by the publisher.
