# Supplementary material for: Determination of the ability of matrix-assisted laser desorption ionization time-of-flight mass spectrometry to identify high-biofilm-producing strains
Source: Front Microbiol. 2023 Jan 10;13:1104405. doi: 10.3389/fmicb.2022.1104405 (PMC9871577; doi:10.3389/fmicb.2022.1104405)
Supplement: Supplementary file 1 [file Data_Sheet_1.PDF]

Table S1. *Staphylococcus aureus* strains included in the study and the identification obtained by predictive models. ID: identifier; abs CV: absorbance value by crystal violet; PLS-DA: Partial Least Squares-Discriminant Analysis; RF: Random Forest.

Strain ID	abs CV	Group	Biofilm production	PLS-DA	Probability (%)	RF	Probability (%)
15.6	1.603	Model	High				
15.15	1.503	Model	High				
13.34	2.593	Model	High				
13.35	1.958	Model	High				
15.36	2.405	Model	High				
15.37	2.125	Model	High				
15.41	1.428	Model	High				
14.45	1.534	Model	High				
15.61	1.514	Model	High				
13.62	3.236	Model	High				
14.82	1.842	Model	High				
14.86	1.376	Model	High				
13.89	1.778	Model	High				
13.97	3.5	Model	High				
15.98	1.595	Model	High				
12.102	1.687	Model	High				
15.104	1.783	Model	High				
12.133	1.54	Model	High				
12.139	2.871	Model	High				
12.157	2.745	Model	High				
15.12	0.457	Model	Low				
14.24	0.3	Model	Low				
13.27	0.308	Model	Low				
15.45	0.231	Model	Low				
14.47	0.453	Model	Low				
14.53	0.421	Model	Low				
14.54	0.182	Model	Low				
14.57	0.057	Model	Low				
14.59	0.107	Model	Low				
14.63	0.387	Model	Low				
15.70	0.363	Model	Low				
14.78	0.315	Model	Low				
12.87	0.063	Model	Low				
12.91	0.378	Model	Low				
12.101	0.06	Model	Low				
12.103	0.266	Model	Low				
12.125	0.157	Model	Low				
12.126	0.204	Model	Low				
12.128	0.399	Model	Low				
12.152	0.432	Model	Low				

12.3	2.101	Validation	High	High	81.93	High	69
13.5	2.142	Validation	High	High	66.56	High	68.83
15.11	1.865	Validation	High	High	88.4	High	81.75
12.13	1.733	Validation	High	High	100	High	79.25
12.16	1.98	Validation	High	High	76.41	High	61
13.21	2.636	Validation	High	High	67.82	High	58.67
15.21	2.946	Validation	High	High	100	High	61.67
15.29	2.862	Validation	High	High	66.53	Low	67.25
15.30	1.543	Validation	High	High	100	High	75.33
15.32	1.503	Validation	High	High	85.88	High	72.33
13.32	2.303	Validation	High	Low	70.43	Low	53.17
12.37	1.805	Validation	High	High	75.66	High	58.25
13.39	1.7	Validation	High	High	59.04	Low	53.5
13.42	1.541	Validation	High	High	53.76	High	66
13.44	3.5	Validation	High	High	100	High	65.42
15.46	1.428	Validation	High	High	74.67	Low	57.08
13.46	3.3	Validation	High	High	64.42	High	80.92
13.48	2.684	Validation	High	Low	54.54	High	56.42
13.50	3.5	Validation	High	High	70.52	High	68.92
12.54	1.65	Validation	High	High	56	High	51.17
12.55	1.895	Validation	High	High	100	High	51.17
13.56	2.77	Validation	High	High	86.23	High	75.33
13.57	1.677	Validation	High	High	76.49	High	68.08
15.58	1.369	Validation	High	High	61.02	High	80.75
15.59	2.004	Validation	High	High	81.91	High	52.75
13.63	2.453	Validation	High	High	83.9	High	74.42
13.67	2.546	Validation	High	Low	52.99	Low	53.08
13.68	2.892	Validation	High	Low	70.97	High	68.83
13.70	2.717	Validation	High	Low	56.81	Low	66.92
13.71	2.996	Validation	High	High	100	High	61.75
12.72	1.493	Validation	High	Low	88.52	Low	63
13.72	1.7	Validation	High	High	56.52	High	82.92
14.73	1.533	Validation	High	High	59.28	High	61.5
14.74	1.531	Validation	High	High	100	High	78.75
13.76	1.586	Validation	High	High	73.28	Low	53.67
12.81	1.508	Validation	High	Low	69.24	High	79.42
15.82	1.539	Validation	High	High	75.38	High	67.17
15.84	1.643	Validation	High	High	100	Low	58.42
15.85	1.561	Validation	High	High	100	Low	57.08
13.86	1.818	Validation	High	High	80.76	High	79.33
14.89	1.64	Validation	High	High	100	Low	60.58
15.94	2.186	Validation	High	High	65.38	High	71.83
15.01	2.602	Validation	High	High	73.26	High	73.58
15.102	2.622	Validation	High	High	100	High	68.75
13.102	2.964	Validation	High	High	89.62	High	75.33

13.103	2.719	Validation	High	Low	62.13	High	70.25
15.103	2.74	Validation	High	Low	68.04	High	65.58
13.104	2.548	Validation	High	High	58.35	High	79.75
13.105	1.526	Validation	High	Low	100	High	53.5
13.106	2.81	Validation	High	High	78.6	Low	51.92
14.107	1.384	Validation	High	High	100	High	66
13.116	2.735	Validation	High	High	93.15	High	61.25
13.119	3.5	Validation	High	High	50.71	High	67.08
13.121	3.037	Validation	High	High	62.75	High	63.25
13.122	3.262	Validation	High	High	60.12	High	65.92
13.124	1.47	Validation	High	Low	58.96	Low	54.75
12.149	2.31	Validation	High	High	90.71	High	90.42
12.151	2.777	Validation	High	High	58.63	High	51
14.2	0.187	Validation	Low	High	95.75	High	86.58
14.5	0.387	Validation	Low	Low	57.46	High	62.42
15.5	0.449	Validation	Low	Low	52.69	High	57.83
15.8	0.425	Validation	Low	Low	83.26	Low	58.67
14.9	0.115	Validation	Low	Low	100	Low	74.08
12.9	0.411	Validation	Low	Low	76.75	Low	73.42
14.12	0.093	Validation	Low	High	53.81	Low	96.92
12.12	0.163	Validation	Low	High	70.03	Low	83
12.18	0.377	Validation	Low	High	78.08	Low	50.58
14.19	0.388	Validation	Low	Low	85.5	Low	69.58
14.20	0.156	Validation	Low	Low	64.05	Low	87.08
14.32	0.368	Validation	Low	Low	78.78	Low	54.83
14.36	0.208	Validation	Low	Low	89.4	High	79.08
13.37	0.475	Validation	Low	Low	92.2	Low	71.33
14.38	0.145	Validation	Low	Low	85.42	Low	79.67
15.44	0.302	Validation	Low	Low	97.02	Low	80.67
12.44	0.474	Validation	Low	Low	66.47	Low	62
13.45	0.291	Validation	Low	Low	56.42	Low	62.42
12.47	0.068	Validation	Low	Low	77.19	Low	87.83
12.48	0.222	Validation	Low	Low	70.86	Low	59.83
14.50	0.052	Validation	Low	Low	84.97	Low	93.08
14.51	0.308	Validation	Low	Low	58.11	High	63.33
12.56	0.075	Validation	Low	Low	70.01	Low	79.42
14.56	0.081	Validation	Low	Low	70.69	High	53.33
12.57	0.175	Validation	Low	Low	79.39	Low	62.25
15.57	0.231	Validation	Low	Low	53.61	Low	60.17
14.58	0.271	Validation	Low	Low	97.51	Low	54.42
14.66	0.303	Validation	Low	Low	76.65	Low	64.75
12.68	0.259	Validation	Low	Low	69.99	Low	66.25
14.68	0.371	Validation	Low	Low	68.27	Low	77.5
12.69	0.373	Validation	Low	High	50.99	High	80.92
15.71	0.167	Validation	Low	Low	69.73	High	54.25

14.71	0.35	Validation	Low	Low	79.07	Low	68.5
14.72	0.067	Validation	Low	Low	82.85	Low	94.42
15.73	0.197	Validation	Low	Low	86.04	Low	78.75
12.75	0.259	Validation	Low	High	55.64	High	83.08
15.75	0.429	Validation	Low	Low	97.39	Low	57.17
12.79	0.029	Validation	Low	Low	59.38	Low	73.33
14.79	0.174	Validation	Low	Low	79.75	High	56.08
15.79	0.403	Validation	Low	Low	83.59	Low	52.25
14.80	0.435	Validation	Low	High	51.03	Low	50.58
12.80	0.459	Validation	Low	Low	63.1	Low	53.5
12.82	0.226	Validation	Low	Low	67.79	Low	58.08
12.88	0.463	Validation	Low	High	77.5	Low	50.83
12.90	0.19	Validation	Low	Low	92.19	Low	83.08
14.92	0.116	Validation	Low	Low	60.06	Low	64.92
15.92	0.267	Validation	Low	High	61.63	Low	56.75
12.93	0.294	Validation	Low	Low	70.38	Low	65
14.94	0.145	Validation	Low	Low	87.83	Low	84.75
14.95	0.085	Validation	Low	High	53.7	Low	69.08
12.96	0.199	Validation	Low	Low	100	Low	85.75
14.98	0.152	Validation	Low	Low	73.66	Low	73.83
12.99	0.356	Validation	Low	High	54.5	High	69.42
14.104	0.238	Validation	Low	Low	51.15	High	71.67
14.105	0.357	Validation	Low	Low	67.94	Low	58.08
12.112	0.23	Validation	Low	Low	50.62	High	70.42
12.114	0.257	Validation	Low	Low	85.49	Low	65.75
12.119	0.424	Validation	Low	Low	67.94	High	64.58
12.122	0.4	Validation	Low	Low	98.72	Low	69.42

Table S2. *Candida albicans* strains included in the study and the identification obtained by predictive models. ID: identifier; abs CV: absorbance value by crystal violet; PLS-DA: Partial Least Squares-Discriminant Analysis; RF: Random Forest.

Strain ID	abs CV	Group	Biofilm production	PLS-DA	Probability (%)	RF	Probability (%)
MT-1	0.156	Model	Low				
MT-101	0.649	Model	Low				
MT-124	0.283	Model	Low				
MT-13	0.55	Model	Low				
MT-14	0.69	Model	Low				
MT-2	0.522	Model	Low				
MT-20	0.548	Model	Low				
MT-26	0.171	Model	Low				
MT-4	0.097	Model	Low				
MT-42	0.707	Model	Low				
MT-44	0.875	Model	Low				
MT-49	0.486	Model	Low				
MT-56	0.578	Model	Low				
MT-61	0.603	Model	Low				
MT-67	0.717	Model	Low				
MT-69	0.366	Model	Low				
MT-7	0.272	Model	Low				
MT-70	0.819	Model	Low				
MT-78	0.784	Model	Low				
MT-8	0.453	Model	Low				
MT-102	1.948	Model	High				
MT-103	1.911	Model	High				
MT-104	1.754	Model	High				
MT-106	1.572	Model	High				
MT-115	2.173	Model	High				
MT-116	1.59	Model	High				
MT-118	1.862	Model	High				
MT-125	1.52	Model	High				
MT-129	2.104	Model	High				
MT-134	1.551	Model	High				
MT-37	1.885	Model	High				
MT-39	2.033	Model	High				
MT-48	1.684	Model	High				
MT-77	1.974	Model	High				
MT-84	1.445	Model	High				
MT-86	1.499	Model	High				
MT-90	1.506	Model	High				
MT-92	1.745	Model	High				
MT-97	1.428	Model	High				

MT-99	1.541	Model	High				
MT-11	0.324	Validation	Low	Low	64.39	High	63
MT-12	0.843	Validation	Low	Low	82.24	Low	95
MT-121	0.316	Validation	Low	Low	62.09	Low	72
MT-130	0.741	Validation	Low	Low	90.25	Low	65
MT-132	0.703	Validation	Low	High	56.38	High	81
MT-16	0.48	Validation	Low	Low	56.44	High	67
MT-21	0.882	Validation	Low	Low	100	Low	83
MT-24	0.921	Validation	Low	Low	54.11	Low	55
MT-29	0.715	Validation	Low	Low	98.11	Low	86
MT-3	0.167	Validation	Low	Low	100	High	76
MT-34	0.245	Validation	Low	Low	88.52	Low	78
MT-35	0.632	Validation	Low	Low	73.42	High	51
MT-38	0.273	Validation	Low	Low	59.8	Low	89
MT-41	0.459	Validation	Low	Low	56.35	High	60
MT-47	0.64	Validation	Low	High	66.97	High	65
MT-5	0.327	Validation	Low	Low	61.8	Low	79
MT-54	0.813	Validation	Low	Low	97.04	Low	67
MT-55	0.912	Validation	Low	Low	87.84	Low	96
MT-57	0.794	Validation	Low	High	93.88	Low	66
MT-58	0.551	Validation	Low	Low	80.04	Low	78
MT-6	0.617	Validation	Low	Low	59.38	Low	84
MT-62	0.672	Validation	Low	Low	55.87	Low	83
MT-65	0.709	Validation	Low	Low	58.61	High	59
MT-66	0.395	Validation	Low	Low	69.56	High	62
MT-68	0.601	Validation	Low	High	50.37	Low	71
MT-75	0.777	Validation	Low	Low	80.73	Low	83
MT-100	1.51	Validation	High	High	51.84	Low	86
MT-105	1.54	Validation	High	Low	100	Low	93
MT-107	1.429	Validation	High	Low	62.33	Low	90
MT-108	1.504	Validation	High	Low	69.05	Low	78
MT-109	1.86	Validation	High	High	71.67	High	65
MT-110	1.92	Validation	High	High	81.26	High	60
MT-113	1.441	Validation	High	Low	68.65	Low	61
MT-114	1.763	Validation	High	High	88.78	High	85
MT-117	2.126	Validation	High	High	100	High	71
MT-120	1.454	Validation	High	Low	70.32	Low	82
MT-123	1.749	Validation	High	High	83.99	High	96
MT-128	1.703	Validation	High	High	67.84	High	82
MT-136	1.89	Validation	High	High	80.62	Low	72
MT-142	1.778	Validation	High	High	97.01	High	92
MT-40	1.932	Validation	High	High	82.24	High	67
MT-59	2.09	Validation	High	High	93.14	High	84
MT-63	1.49	Validation	High	High	51.54	Low	56
MT-74	1.593	Validation	High	Low	62.24	Low	86

MT-79	1.543	Validation	High	Low	93.43	Low	95
MT-80	1.542	Validation	High	Low	70.88	Low	55
MT-81	1.613	Validation	High	High	92.22	High	93
MT-83	1.589	Validation	High	High	82.72	Low	57
MT-87	1.864	Validation	High	High	61.78	High	64
MT-95	1.951	Validation	High	High	100	Low	57
MT-96	1.536	Validation	High	Low	99.46	Low	66
